# Utility of conventional clinical risk scores in a low-risk COVID-19 cohort

**DOI:** 10.1186/s12879-021-06768-3

**Published:** 2021-10-24

**Authors:** Jinghao Nicholas Ngiam, Nicholas W. S. Chew, Sai Meng Tham, Zhen Yu Lim, Tony Y. W. Li, Shuyun Cen, Paul Anantharajah Tambyah, Amelia Santosa, Ching-Hui Sia, Gail Brenda Cross

**Affiliations:** 1 Department of Medicine, National University Health System, Singapore, Singapore; 2 Department of Cardiology, National University Heart Centre Singapore, Singapore, Singapore; 3 Department of Infectious Diseases, National University Health System, 1E Kent Ridge Rd, NUHS Tower Block, Level 10, Singapore, 119228 Singapore; 4 Department of Rheumatology, National University Health System, Singapore, Singapore; 5 Department of Medicine, Yong Loo Lin School of Medicine, National University of Singapore, Singapore, Singapore; 6 Infectious Diseases Translational Research Programme, Department of Medicine, Yong Loo Lin School of Medicine, National University of Singapore, Singapore, Singapore

**Keywords:** COVID-19, Risk score, Outcomes, Fever, Singapore

## Abstract

**Background:**

Several specific risk scores for Coronavirus disease 2019 (COVID-19) involving clinical and biochemical parameters have been developed from higher-risk patients, in addition to validating well-established pneumonia risk scores. We compared multiple risk scores in predicting more severe disease in a cohort of young patients with few comorbid illnesses. Accurately predicting the progression of COVID-19 may guide triage and therapy.

**Methods:**

We retrospectively examined 554 hospitalised COVID-19 patients in Singapore. The CURB-65 score, Pneumonia Severity Index (PSI), ISARIC 4C prognostic score (4C), CHA_2_DS_2_-VASc score, COVID-GRAM Critical Illness risk score (COVID-GRAM), Veterans Health Administration COVID-19 index for COVID-19 Mortality (VACO), and the “rule-of-6” score were compared for three performance characteristics: the need for supplemental oxygen, intensive care admission and mechanical ventilation.

**Results:**

A majority of patients were young (≤ 40 years, n = 372, 67.1%). 57 (10.3%) developed pneumonia, with 16 (2.9% of study population) requiring supplemental oxygen. 19 patients (3.4%) required intensive care and 2 patients (0.5%) died. The clinical risk scores predicted patients who required supplemental oxygenation and intensive care well. Adding the presence of fever to the CHA_2_DS_2_-VASc score and 4C score improved the ability to predict patients who required supplemental oxygen (c-statistic 0.81, 95% CI 0.68–0.94; and 0.84, 95% CI 0.75–0.94 respectively).

**Conclusion:**

Simple scores including well established pneumonia risk scores can help predict progression of COVID-19. Adding the presence of fever as a parameter to the CHA_2_DS_2_-VASc or the 4C score improved the performance of these scores in a young population with few comorbidities.

**Supplementary Information:**

The online version contains supplementary material available at 10.1186/s12879-021-06768-3.

## Background

The pandemic of Coronavirus disease 2019 (COVID-19) has left the entire globe reeling, with almost 2 million lives lost worldwide as of February 2021, and an economic and social fall-out that is yet to be fully realised [1]. Despite the shocking amount of death and morbidity from this disease, it is estimated up to 80% of patients have mild disease or are asymptomatic, with 14% having severe disease including pneumonia and the remaining 5% with critical illness including multi-organ failure (MOF), septic shock, respiratory failure which can progress to death [2]. Amongst those with moderate to critical illness, the natural history of the disease is such that patients first develop a respiratory illness, typically with a fever, which then progresses in the second week of illness to the development of a pulmonary infiltrate, and in a proportion, this then progresses to the development of acute respiratory distress syndrome (ARDS) and MOF [3]. Risk scores developed and utilised thus far have focused on predicting which patients who develop severe to critical disease and would benefit from intensive care therapies [4–6].

It may, however, be more useful to discover a risk score which can predict those who progress to needing the earlier stage of oxygenation, when the initiation of COVID-19 specific therapies have more utility. Therapies such as dexamethasone and intravenous remdesivir have been shown to confer survival benefit in patient groups with moderate to severe disease which requires supplemental oxygen [7–9]. Thus far the COVID-19 specific risk scores developed require several laboratory parameters or complicated clinical parameter calculations [10–13], yet a simple tool that did not require laboratory parameters would be of greater utility particularly in an emergency room or clinic setting, especially in low and middle income countries.

## Methods

We examined the first 554 confirmed consecutive patients with reverse transcriptase-polymerase chain reaction (RT-PCR) proven COVID-19 admitted to a single tertiary institution in Singapore from 23rd January 2020 to 30th April 2020. No patients were lost to follow-up or excluded from the analyses.

We retrospectively collected data on the demographic background (age, gender), medical co-morbidities, clinical profile (symptom status and clinical presentation), baseline haematological and biochemical parameters within 24 h of admission for each patient. Clinical outcomes were also examined during each patient’s hospital stay, including the development of pneumonia, need for supplemental oxygen, need for intensive care, mechanical ventilation and mortality. We have published the demographic profile of this cohort previously and shown that there was a shift from an older, local population with more comorbidities who were at risk of severe disease to a young, migrant worker population with few comorbidities who largely had mild disease [14]. Having noticed that age was a common variable identified in all the risk scores examined, in this current study, we present the analysis of the same cohort stratified within three age bands (40 years or less, 41–64 years of age and those greater than 65 years of age). The age cut-off of 40 was defined by Singapore’s Ministry of Health (based on unpublished aggregate data from Singapore COVID-19 cases) to identify those who were at higher risk of severe COVID-19 illness, and who thus required isolation for the first week of illness in hospitals, rather than in community isolation facilities. Later on, this cut-off was lowered further to 30 years of age [15]. Globally, the age group of patients below 40 has also been found to have the lowest mortality as well [16]. The age of 65 was used as a second cut-off since mortality risk increased significantly at and above this age [17].

For each patient, we tabulated information on medical-comorbidities, clinical disease presentation, laboratory results at baseline and clinical outcomes and each of 7 clinical risk scores. We compared risk scores that had been designed in community acquired pneumonia, such as the CURB-65 score [5], and pneumonia severity index [6], as well as scores specifically derived in the context of COVID-19, such as the 4C score [10], “rule-of-6” score [11], VACO score [12], and COVID-GRAM score [13]. In addition, we also examined the CHA_2_DS_2_-VASc score [4], which has been demonstrated to correlate with adverse outcomes in COVID-19 cohorts [18, 19]. Day of illness refers to the number of days post illness onset to hospital admission. Pneumonia was defined as the presence of an infiltrate seen a chest radiograph or computed tomography. Persistent fever was defined as a fever ≥ 72-h, to define the subset amongst our low-risk cohort with greater risk of severe illness, as previously shown [3]. Thus, we added the presence of fever (1 point) to the CHA_2_DS_2_-VASc score and 4C score (existing clinical risk scores), and sought to explore if this would improve the ability score to predict adverse outcomes.

To compare the three age categories, one-way analysis of variance (ANOVA) was used for continuous parameters, with the data presented as mean (± standard deviation). Kruskal–Wallis or Chi-squared tests for association (where appropriate) were used for categorical parameters, with the data presented as frequencies and percentages. Risk scores for the overall potential and each age category was presented as median score with the corresponding interquartile range. Performance of each risk score in identifying patients who required supplemental oxygen, intensive care admission and mechanical ventilation were assessed by means of area of receiver operating characteristic curve (AUC).

A p-value of less than 0.05 was considered significant. Data analysis was done on SPSS version 20.0 (SPSS, Inc., Chicago, Illinois). Ethics approval was obtained from the hospital’s institutional review board (National Healthcare Group (NHG) Domain Specific Review Board (DSRB) 2020/00545) prior to its conduct. Data collected was anonymised and a waiver of informed consent was obtained. Study methods were carried out in accordance with guidelines and regulations by the Declaration of Helsinki and DSRB.

## Results

Of the 554 consecutive patients diagnosed with COVID-19 examined, the majority (n = 372, 67.1%) were ≤ 40 years old, 30.0% (n = 166) were 40–64 years old and the remaining 2.9% (n = 16) were ≥ 65 years old. Most patients did not have medical comorbidities. Patients ≤ 40 years old were also more likely to be asymptomatic when compared with those aged 41–64 and ≥ 65 years old (14.0% asymptomatic compared with 8.4% and 0.0% respectively). Patients ≥ 65 years old tended to present later in their illness, with a longer duration of fever and with lower oxygen saturations (Table 1). The proportion of patients with radiographic evidence of pneumonia was highest in the oldest patients (≥ 65 years, n = 8, 50.0%), when compared with those aged 41–64 (19.9%) and ≤ 40 years old (4.3%). With increasing age, patients were also more likely to require supplemental oxygen (up to 12.5% in those ≥ 65 years old), intensive care and mechanical ventilation (up to 31.3% in those ≥ 65 years old). The two deaths in our cohort both occurred in patients ≥ 65 years of age (Table 1).

**Table 1:** Clinical profile of hospitalized patients with COVID-19 by three age categories

Parameter	Overall (n = 554)	Age ≤ 40 years (n = 372)	Age 41 to 64 years (n = 166)	Age ≥ 65 years (n = 16)	p-value
Age (years)	37 (± 12)	30 (± 5)	49 (± 6)	71 (± 6)	< 0.001
Gender (Male)	477 (86.9%)	327 (88.9%)	139 (83.7%)	12 (75.0%)	0.096
Medical co-morbidities					
Prior history of hypertension	53 (12.3%)	8 (2.8%)	34 (25.2%)	11 (68.8%)	< 0.001
Prior history of hyperlipidaemia	34 (8.1%)	2 (0.7%)	20 (15.6%)	12 (75.0%)	< 0.001
Prior history of diabetes mellitus	21 (5.1%)	3 (1.1%)	14 (11.2%)	4 (25.0%)	< 0.001
Prior history of asthma	6 (1.5%)	4 (1.4%)	2 (1.7%)	0 (0.0%)	0.946
Clinical profile					
Asymptomatic illness	66 (11.9%)	52 (14.0%)	14 (8.4%)	0 (0.0%)	0.061
Day of illness at presentation	3.5 (± 5.1)	3.1 (± 5.0)	4.0 (± 5.1)	6.8 (± 5.0)	0.005
Length of days with fever	1.2 (± 2.4)	1.0 (± 2.2)	1.6 (± 2.5)	2.7 (± 4.3)	0.001
Admission temperature (°C)	37.7 (± 3.9)	37.5 (± 0.8)	37.5 (± 0.9)	37.5 (± 0.6)	0.779
Systolic blood pressure (mmHg)	130 (± 17)	127 (± 16)	136 (± 19)	131 (± 19)	< 0.001
Diastolic blood pressure (mmHg)	81 (± 12)	81 (± 11)	83 (± 14)	69 (± 10)	< 0.001
Oxygen saturation (%)	98 (± 3)	98 (± 1)	98 (± 3)	94 (± 9)	< 0.001
Pulse rate (per min)	94 (± 19)	95 (± 19)	93 (± 19)	88 (± 12)	0.202
Respiratory rate (per min)	19 (± 7)	19 (± 6)	19 (± 7)	21 (± 6)	0.378
Baseline laboratory investigations					
Total White Cell Count (× 10^9^/L)	6.5 (± 2.2)	6.5 (± 1.9)	6.4 (± 2.6)	6.7 (± 3.5)	0.810
Absolute neutrophil count (× 10^9^/L)	4.18 (± 7.9)	4.20 (± 9.47)	4.07 (± 2.76)	4.79 (± 3.29)	0.937
Absolute lymphocyte count (× 10^9^/L)	1.90 (± 2.02)	2.01 (± 2.35)	1.72 (± 0.97)	1.18 (± 0.43)	0.106
Haemoglobin (g/dL)	14.9 (± 1.6)	15.1 (± 1.5)	14.4 (± 1.6)	13.0 (± 2.3)	< 0.001
Platelet Count (× 10^9^/L)	228 (± 60)	231 (± 29)	221 (± 62)	225 (± 71)	0.200
Sodium (mmol/L)	138 (± 3)	138 (± 2)	138 (± 3)	134 (± 5)	0.076
Urea (mmol/L)	3.9 (± 2.6)	3.7 (± 2.7)	4.0 (± 1.5)	7.7 (± 5.5)	< 0.001
Creatinine (mmol/L)	79 (± 30)	77 (± 15)	81 (± 46)	108 (± 61)	< 0.001
AST (units/L)	38 (± 48)	37 (± 25)	40 (± 81)	50 (± 28)	0.543
ALT (units/L)	46 (± 44)	48 (± 40)	41 (± 52)	37 (± 24)	0.234
LDH (units/L)	436 (± 423)	412 (± 408)	477 (± 462)	630 (± 279)	0.087
C-reactive protein (mg/L)	14 (± 27)	10. (± 23)	19 (± 29)	65 (± 61)	< 0.001
Ferritin (ug/L)	179 (± 216)	139 (± 91)	263 (± 348)	438 (± 383)	< 0.001
Clinical outcomes					
Pneumonia	57 (10.3%)	16 (4.3%)	33 (19.9%0	8 (50.0%)	< 0.001
Requiring supplemental oxygen	16 (2.9%)	6 (1.6%)	8 (4.8%)	2 (12.5%)	0.008
Persistent fever > 72 h	40 (7.3%)	16 (4.3%)	21 (12.7%)	3 (20.0%)	< 0.001
Acute kidney injury	45 (8.1%)	23 (6.2%)	13 (7.8%)	9 (56.3%)	< 0.001
Required intensive care monitoring	19 (3.4%)	3 (8.8%)	11 (6.7%)	5 (31.3%)	< 0.001
Required mechanical ventilation	16 (2.9%)	2 (0.5%)	9 (5.4%)	5 (31.3%)	< 0.001
Myocarditis/myocardial injury	3 (0.7%)	0 (0.0%)	2 (1.6%)	1 (20.0%0	< 0.001
Death	2 (0.5%)	0 (0.0%)	0 (0.0%)	2 (33.3%)	< 0.001
Clinical risk scores					
CURB-65 Score (median, interquartile range)	0 (0–0)	0 (0–0)	0 (0–0)	1 (1–2)	< 0.001
Pneumonia Severity Index (median, interquartile range)	35 (28–45)	30 (27–35)	39 (42–53)	68 (65–75)	< 0.001
CHA_2_DS_2_-VASc Score (median, interquartile range)	0 (0–0)	0 (0–0)	0 (0–1)	1 (1–2)	< 0.001
COVID-GRAM Score (%)	0.0218 (0.0123–0.500)	0.0153 (0.0112–0.500)	0.0636 (0.0204–0.500)	0.500 (0.500–0.500)	< 0.001
VACO Score (%)	0.00403 (0.00403–0.500)	0.0153 (0.0112–0.500)	0.0636 (0.004–0.5000)	0.500 (0.182–0.500)	< 0.001
4C Score	2 (1–3)	1 (1–2)	2 (1–3)	3 (2–6)	< 0.001
“Rule-of-6” Score	0 (0–0)	0 (0–0)	0 (0–0)	2 (1–2)	< 0.001

AST: aspartate transaminase; ALT: alanine transaminase; LDH: lactate dehydrogenase; CURB-65: (confusion, blood urea > 42,8 mg/dl, respiratory rate > 30/min, blood pressure < 90/60 mm Hg, age > 65); CHA_2_DS_2_-VASc: Congestive heart failure, Hypertension, Age ≥ 75 years (doubled), Diabetes mellitus, Prior Stroke or TIA or thromboembolism (doubled), Vascular disease, Age 65–74 years, Sex category; HF: heart failure; LV: left ventricular; MI: myocardial infarction; PAD: peripheral artery disease; TE: thromboembolism; TIA: transient ischemic attack. VACO: Veterans Health administration COVID-19; 4C: Coronavirus Clinical Characterization Consortium

**Fig. 1:**
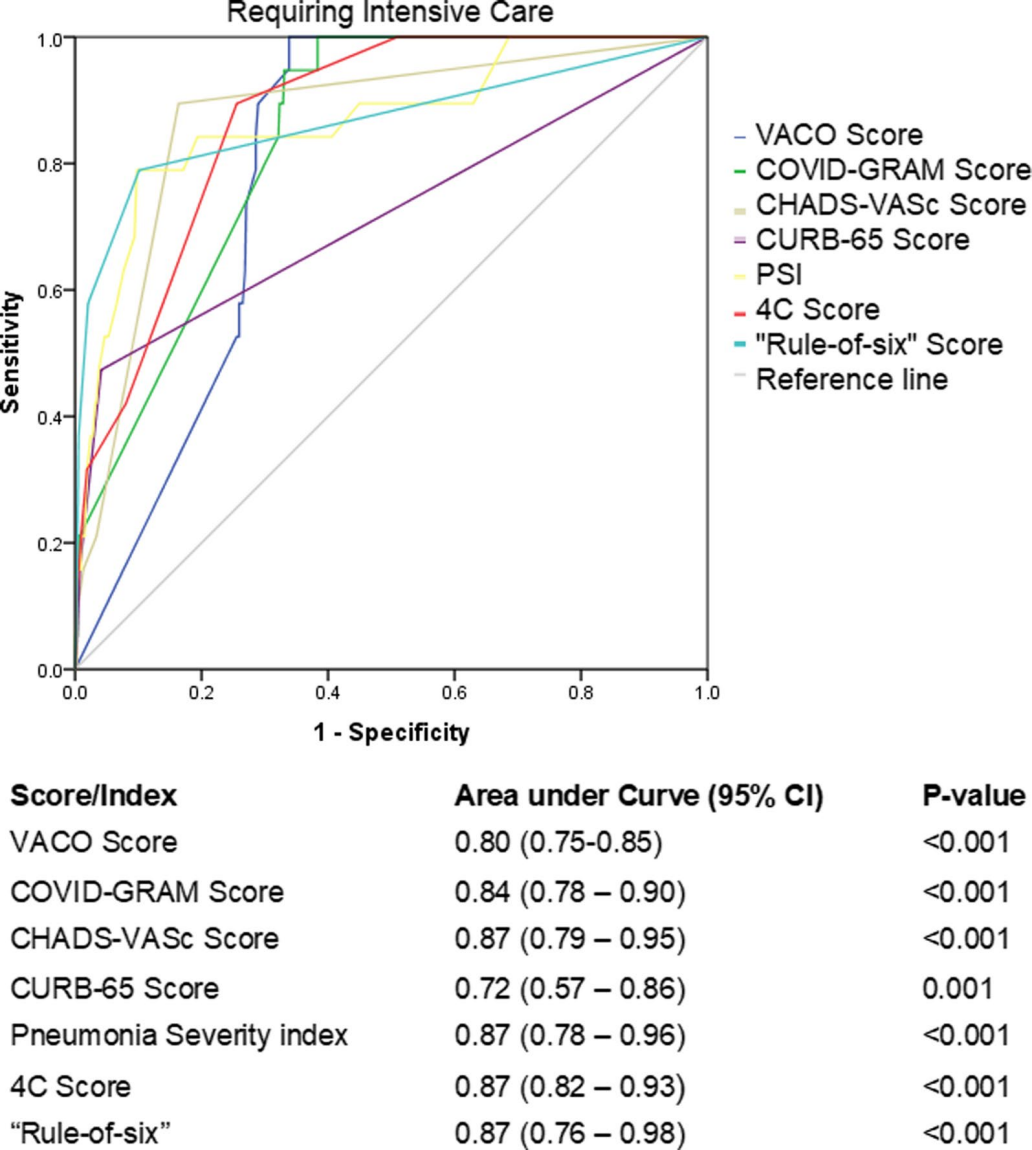
Receiver operating characteristic curve of clinical risk scores in predicting need for intensive care

The median score (with interquartile range) for each age category is shown in Table 1. Of the 7 scores studied, 4C Score, Rule-of-6, PSI and CHA_2_DS_2_-VASc Score all had an AUC of 0.87 in predicting patients who required an ICU admission, and the CURB-65 score performed the poorest with an AUC of 0.72 (95% CI 0.57–0.86) (Fig. 1).

**Fig. 2:**
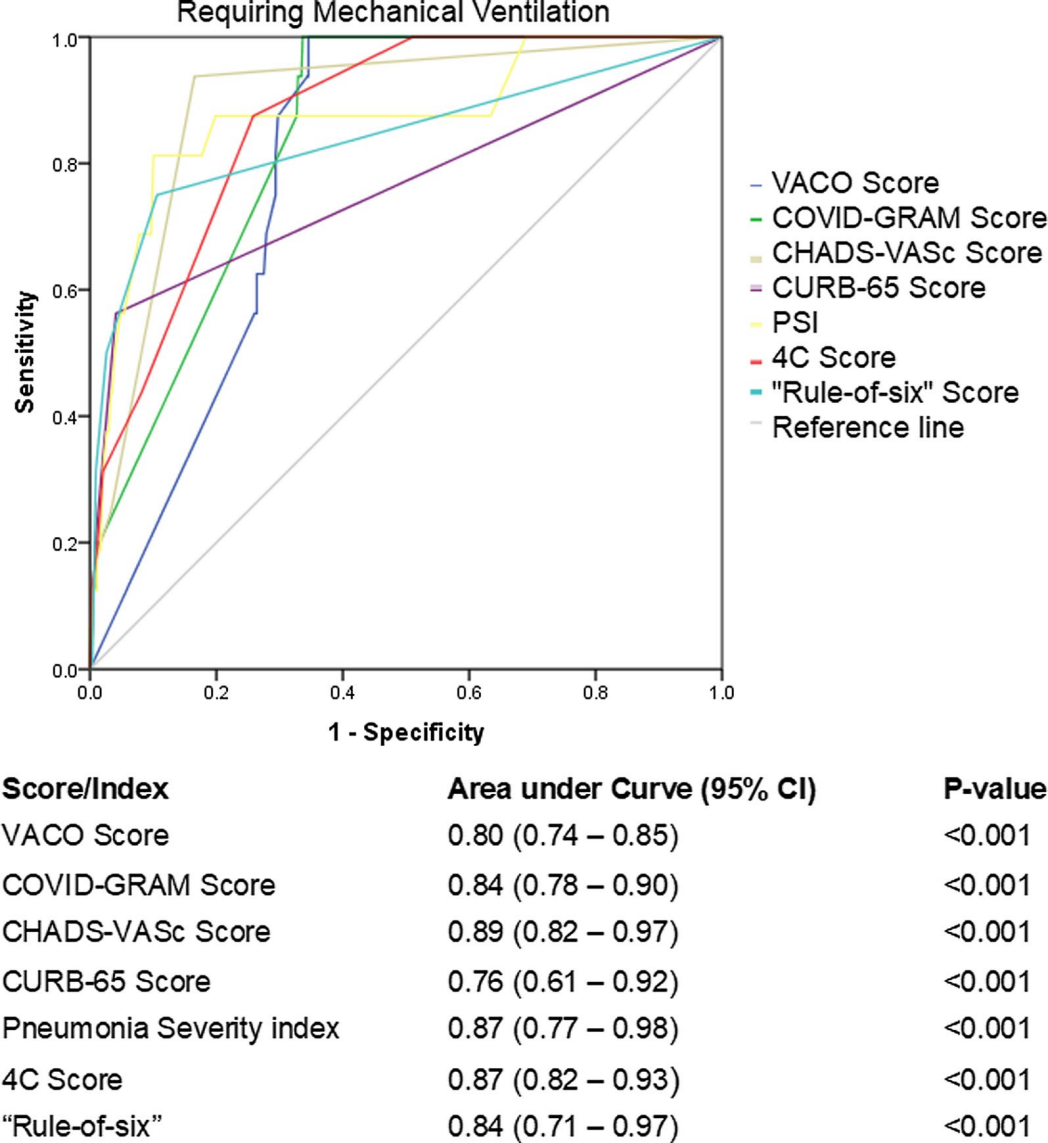
Receiver operating characteristic curve of clinical risk scores in predicting need for mechanical ventilation

In predicting the risk of requiring mechanical ventilation, the CHA_2_DS_2_-VASc Score performed the best with an AUC of 0.89 (95% CI 0.82–0.97), with other scores ranging between an AUC of 0.80 to 0.87, however once again the CURB-65 score performed poorly with an AUC of 0.76 (95% CI 0.61–0.92, Fig. 2).

**Fig. 3:**
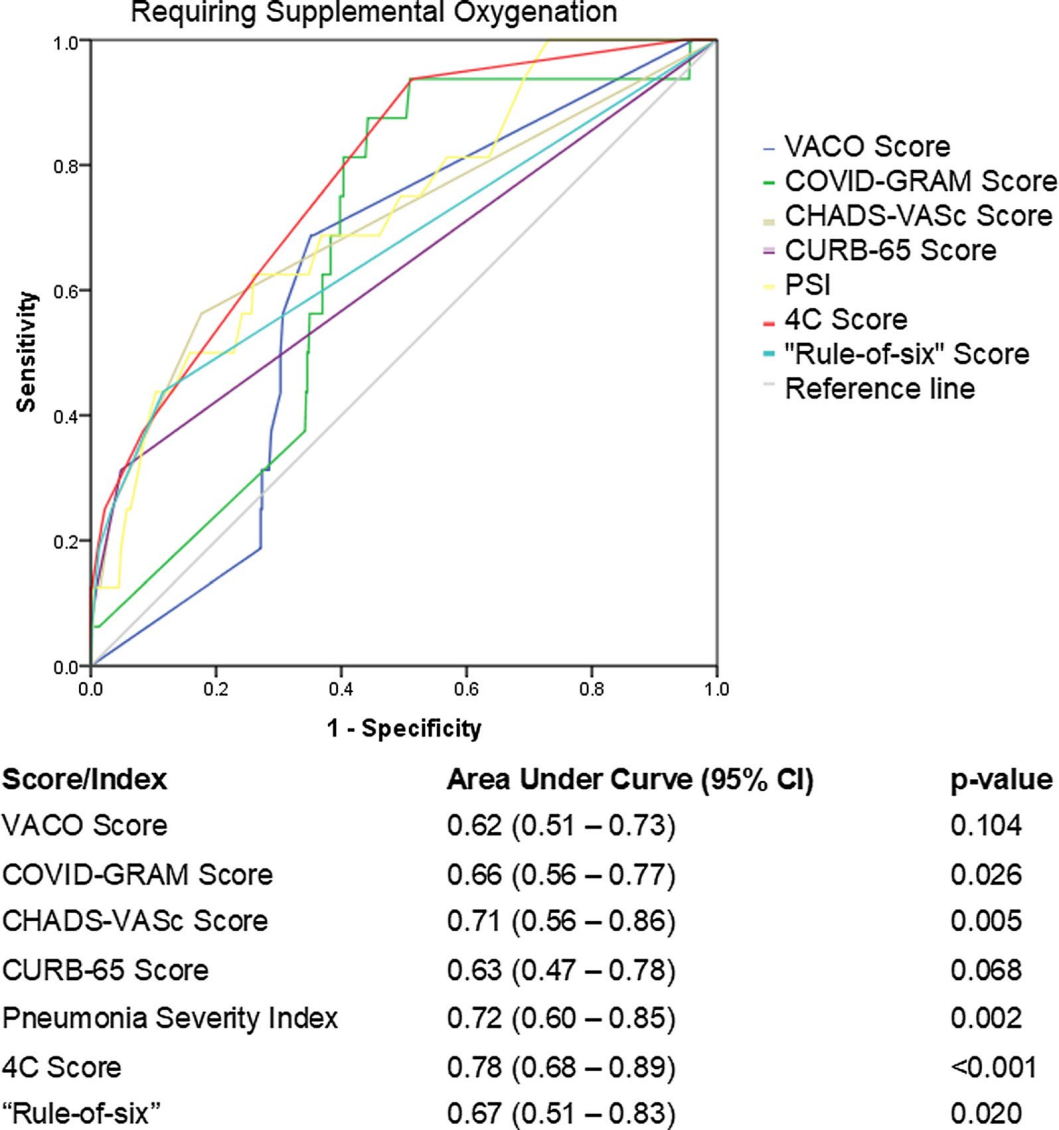
Receiver operating characteristic curve of clinical risk scores in predicting need for supplemental oxygen

**Fig. 4:**
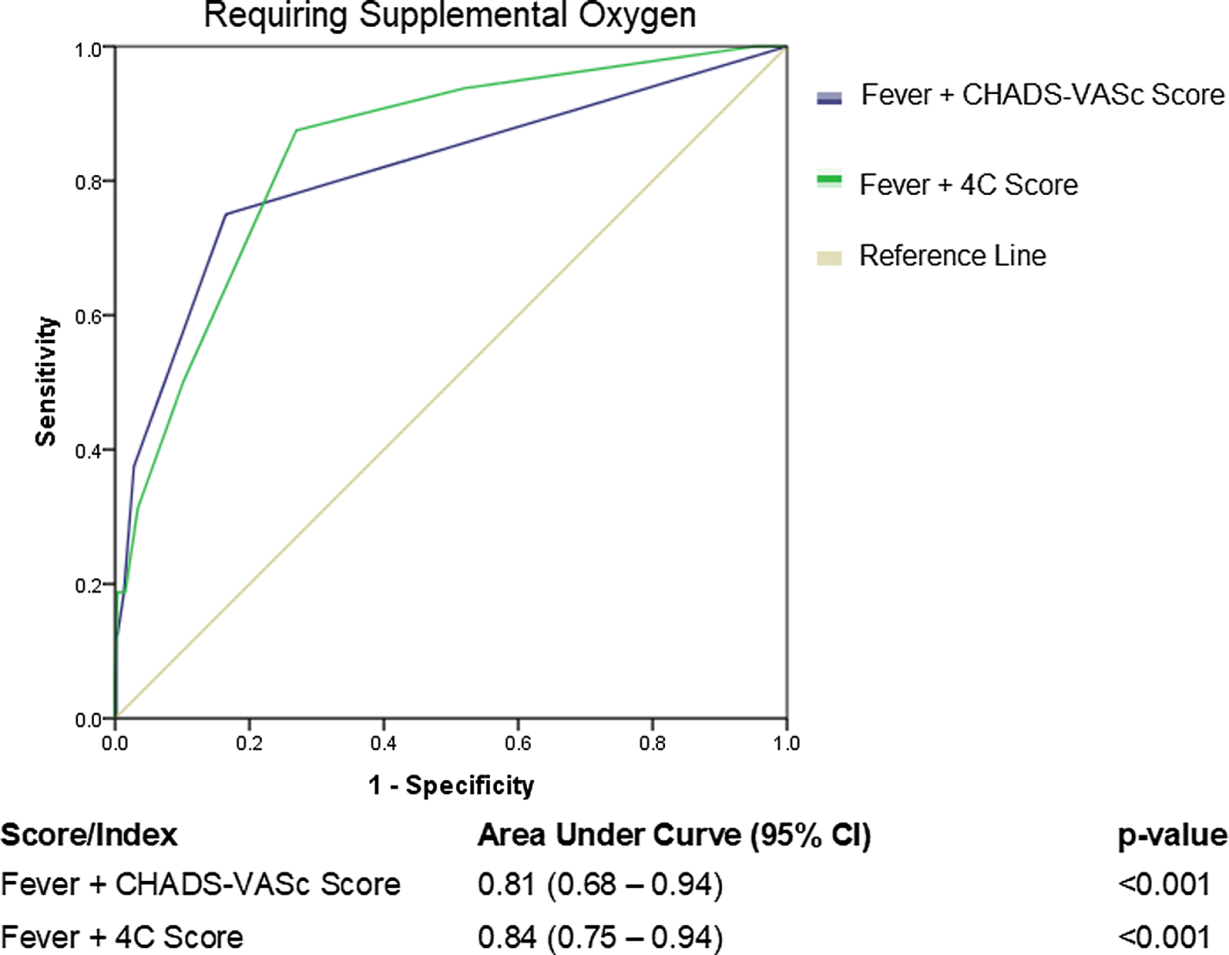
Receiver operating characteristic curve shows improved performance with the addition of the presence of fever in two clinical risk scores predicting need for supplemental oxygen

Finally, all scores, comparatively, underperformed in predicting patients who required supplemental oxygen. The best performing score was the 4C score with an AUC 0.78 (95% CI 0.68–0.89, p < 0.001), with 4 other scores meeting the threshold for statistical significance: PSI AUC 0.72 (95% CI 0.60–0.85, p = 0.002); CHA_2_DS_2_-VASc Score AUC 0.71 (95% CI 0.56–0.86, p = 0.005); “Rule-of-6” score AUC 0.67 (95% CI 0.51–0.83, p = 0.020); COVID-GRAM AUC 0.66 (95% CI 0.56–0.77, p = 0.026). VACO and CURB-65 scores did not meet statistical significance (Fig. 3). Adding the presence of fever (1 point) to existing scores such as the CHA_2_DS_2_-VASc Score and 4C score improved the ability of the scores to predict patients who required supplemental oxygen (CHA_2_DS_2_-VASc AUC 0.81 (95% CI 0.68–0.94) and 4C AUC 0.84 (95% CI 0.75–0.94) respectively) (Fig. 4).

## Discussion

Large and repeated outbreaks of COVID-19 continue to place a strain on healthcare institutions and intensive care facilities [10]. A simple and effective clinical risk score could prove to be invaluable in determining which patients with COVID-19 in the community are likely to require oxygen supplementation and therefore would benefit from monitoring in hospital and the initiation of COVID-19 specific therapeutics as early as when they first require oxygen supplementation, i.e. at the juncture of disease severity when they are most likely to benefit [20]. Yet such a definitive tool for COVID-19, that does not require complex laboratory tests, remains elusive [21].

We evaluated 4 risk scores which were developed to predict risk of critical illness or death specifically in patients with COVID-19. This includes the COVID-GRAM score, developed and validated in large Chinese cohorts of patients [13], the 4C score was derived and validated by cohorts in the United Kingdom [10], the VACO score [12], validated in multiple large American cohorts [22], and finally the Rule-of -6 score developed from a small cohort in Singapore [11]. Of these 4 scores, all required laboratory tests or radiography or both, except for the VACO score, which incorporates the Charlson Comorbidity Index [23], a complex index which requires substantial knowledge of a patient’s past medical history. Additional file 1: Table S1 shows the complexity of the score calculation across the risk scores analysed in this study.

Additionally, we evaluated three non-COVID specific tests. The CHA_2_DS_2_-VASc score was originally developed to describe the risk of stroke in patients with atrial fibrillation [4], was the only score tested which was not developed for a pneumonic illness, and also did not require either laboratory tests or radiography. The score has previously been shown to be associated with mortality in hospitalised patients with COVID-19 in two other cohorts, including the current cohort of patients [18, 19].

The performance of the evaluated scores revealed that all scores performed well in predicting the severe end of the disease spectrum of COVID-19. This is not unsurprising given that most of these scores were developed to predict mortality or critical illness, the risks factors of which are largely consistent across disease spectrums and even epidemiological cohorts. The CHA_2_DS_2_-VASc score performed well in predicting those patients at risk of ICU admission and also those who were at risk of mechanical ventilation. The CHA_2_DS_2_-VASc score is note-worthy since it does not require any biochemical markers or radiography, and requires basic knowledge of cardiovascular medical history, along with age and sex. The presence of hypertension, diabetes, congestive cardiac failure, previous ischaemic heart disease, and stroke which increase the CHA_2_DS_2_-VASc score have been strongly associated with adverse outcomes in patients with COVID-19 [24].

Indeed, even after adjusting for age, the presence of hypertension as a comorbidity had been one of the earliest identified risk factors for severe COVID-19 illness [25]. Additionally patients with diabetes mellitus have consistently had poorer outcomes in COVID-19 illness [26]. Hyperglycaemia at presentation with underlying poor diabetes control has been shown to be associated with a four-fold increase in mortality compared with those with normoglycaemia [27]. Patients with existing ischaemic heart disease were more likely to present with elevated troponins during their COVID-19 illness, suggesting the presence of myocardial injury, which has been independently associated with adverse clinical outcomes and mortality [28]. The predictive value of the CHA_2_DS_2_-VASc score, might lie in the unique pathology associated with critical COVID-19 disease wherein vascular phenomena are thought to contribute substantially to multi-organ failure and ultimately death [29].

All scores performed less well in predicting those who needed supplemental oxygen therapy. The best performing score in this regard was the 4C, which requires both urea, C-reactive protein, a lymphocyte count as well as a Chest-X-ray to determine the presence of pneumonia. The PSI and the CHA_2_DS_2_-VASc score were found to have AUCs of 0.72 and 0.71 respectively in predicting risk to needing supplemental oxygen, but with relatively wide confidence intervals, which might undermine their performance in some instances. However, adding the presence or absence of fever, a parameter that is readily available in nearly all patients, to the CHA_2_DS_2_-VASc and the 4C score significantly improved the ability of the scores to predict patients who required supplemental oxygenation.

We have previously shown that both the CHA_2_DS_2_-VASc score and the presence of fever individually were associated with adverse outcomes in COVID-19 [3, 18, 19]. Here we show that when we combine the both to develop a composite score, it better predicts the need for supplemental oxygenation, even in our relatively low-risk cohort of hospitalised patients with COVID-19.

## Limitations

Our study was based on the experience of a single-centre, moderately-sized cohort of patients diagnosed with COVID-19 in Singapore. Risk scores were tabulated based on information at the time of hospital admission and we did not have access to include data from a small group of patients who were assessed in the emergency department were deemed not to require hospitalisation, and were managed in isolation facilities in the community. It would be important for the findings from our study, namely the performance of the CHA_2_DS_2_-VASc and fever be evaluated in more diverse epidemiological cohorts and in different economies worldwide.

## Conclusions

We evaluated 7 risk scores in patients with COVID-19 to identify those at risk of critical illness or supplemental oxygen. Most require laboratory investigations and may be difficult to apply in an outpatient setting or for immediate triage. We found that both simple scores as well as more well-established disease risk scores can help predict progression of COVID-19. Adding the presence of fever as a parameter to the CHA_2_DS_2_-VASc or the 4C score improved the performance of these scores in predicting adverse outcomes in relatively young patients with few medical co-morbidities.

## Supplementary Information

**Additional file 1: Supplementary Table S1.** Components of commonly used risk scores in the context of COVID-19 illness.

## Data Availability

Data may be made available on reasonable request from the corresponding author.

## Abbreviations

4C: ISARIC 4C prognostic score
ANOVA: Analysis of variance
ARDS: Acute respiratory distress syndrome
AUC: Area under receiver operating characteristic curve
COVID-19: Coronavirus disease 2019
DSRB: Domain-Specific Review Board
ICU: Intensive care unit
MOF: Multi-organ failure
NHG: National Healthcare Group, Singapore
PSI: Pneumonia severity index
RT-PCR: Reverse transcriptase polymerase chain reaction
VACO: Veteran’s Health Administration COVID-19 index for COVID-19 mortality
