# Supplementary material for: Utility of conventional clinical risk scores in a low-risk COVID-19 cohort
Source: BMC Infect Dis. 2021 Oct 24;21:1094. doi: 10.1186/s12879-021-06768-3 (PMC8542362; doi:10.1186/s12879-021-06768-3)
Supplement: Supplementary file 1 — Additional file 1: Supplementary Table S1. Components of commonly used risk scores in the context of COVID-19 illness. [file 12879_2021_6768_MOESM1_ESM.docx]

**Supplementary Table 1**

| **Risk Score** | **Components of Score** | **Predicted outcome** | **COVID-19 validation** |
| --- | --- | --- | --- |
| VACO Score^a^ | Clinical profile:  Age  Sex  Past Medical History - Charlson Comorbidity Index | Risk of 30-day Mortality after COVID-19 infection | Validated in US cohort^a^ |
| COVID-GRAM Score^b^ | Clinical profile:  Age  Haemoptysis  Dyspnoea  Unconsciousness  Number of comorbidities  Cancer history  Laboratory tests:  Neutrophil-lymphocyte ratio (NLR)  Lactate dehydrogenase  Direct bilirubin  Imaging:  X-ray abnormality | Risk of critical illness (intensive care, mechanical ventilation or death) in hospitalised patients with COVID-19 | Validated in Chinese cohort^b^ |
| 4C Score^c^ | Clinical profile:  Age  Sex  Number of comorbidities  Respiratory rate  Peripheral oxygenation saturation on room air  Glasgow coma scale  Laboratory tests:  Urea  C-reactive protein | Risk of in-hospital mortality in hospitalised COVID-19 patients | Validated in UK cohort^c^ |
| “Rule-of-6”^d^ | Clinical profile:  Age  Laboratory tests: C-reactive protein  Ferritin  Lactate dehydrogenase | Requiring oxygen, intensive care, intubation | Validated in Singapore cohort^d^ |
| CHA_2_DS_2_-VASc Score^e^ | Clinical profile:  Age  Sex  Congestive cardiac failure history  Hypertension history  Stroke/Transient ischemic attack/thromboembolism history  Vascular disease history (Prior myocardial infarction, peripheral vascular disease, or aortic plaque)  Diabetes history | Risk of stroke in patients with atrial fibrillation | Validated in 2 cohorts: Asia and US^f^ |
| CURB-65 Score^g^ | Confusion  Urea  Respiratory rate  Blood pressure  Age | Risk of mortality from community-acquired pneumonia to determine inpatient versus outpatient treatment. | Validated widely^h^ |
| Pneumonia Severity Index^i^ | Clinical profile:  Age  Sex  Nursing home resident  Neoplastic disease  Liver disease history  Congestive heart failure history  Cerebrovascular disease history  Renal disease history  Altered mental status  Respiratory rate  Systolic blood pressure  Temperature  Pulse rate  Laboratory tests:  pH  Urea  Sodium  Glucose  Haematocrit  Partial pressure of oxygen  Imaging:  Pleural effusion on X-ray | Risk of mortality from community-acquired pneumonia | Validated widely^j^ |

^a^ King JT Jr, Yoon JS, Rentsch CT, Tate JP, Park LS, Kidwai-Khan F, Skanderson M, Hauser RG, Jacobson DA, Erdos J, Cho K, Ramoni R, Gagnon DR, Justice AC. Development and validation of a 30-day mortality index based on pre-existing medical administrative data from 13,323 COVID-19 patients: The Veterans Health Administration COVID-19 (VACO) Index. PLoS One. 2020 Nov 11;15(11):e0241825. doi: 10.1371/journal.pone.0241825. PMID: 33175863; PMCID: PMC7657526.

^b^ Liang W, Liang H, Ou L, Chen B, Chen A, Li C, Li Y, Guan W, Sang L, Lu J, Xu Y, Chen G, Guo H, Guo J, Chen Z, Zhao Y, Li S, Zhang N, Zhong N, He J; China Medical Treatment Expert Group for COVID-19. Development and Validation of a Clinical Risk Score to Predict the Occurrence of Critical Illness in Hospitalized Patients With COVID-19. JAMA Intern Med. 2020 Aug 1;180(8):1081-1089. doi: 10.1001/jamainternmed.2020.2033. PMID: 32396163; PMCID: PMC7218676.

^c^ Knight SR, Ho A, Pius R, et al. Risk stratification of patients admitted to hospital with covid-19 using the ISARIC WHO Clinical Characterisation Protocol: development and validation of the 4C Mortality Score. BMJ. 2020;370:m3339.

^d^ Dickens BSL, Lim JT, Low JW, Lee CK, Sun Y, Nasir HBM, Mohamed Akramullah FA, Yan G, Oon J, Yan B, Sun L, Cook AR, Tambyah PA, Chai LYA. Simple “Rule-of-6” Predicts Severe Coronavirus Disease 2019 (COVID-19). Clinical Infectious Diseases, 2020;ciaa938, https://doi.org/10.1093/cid/ciaa938

^e^ Lip GY, Nieuwlaat R, Pisters R, Lane DA, Crijns HJ. Refining clinical risk stratification for predicting stroke and thromboembolism in atrial fibrillation using a novel risk factor-based approach: the euro heart survey on atrial fibrillation. Chest. 2010 Feb;137(2):263-72. doi: 10.1378/chest.09-1584. Epub 2009 Sep 17. PMID: 19762550.

^fi^ Chew N, Ngiam N, Sia CH. The Utility of CHA(2)DS(2)-VASc Scores as a Risk Assessment Tool in Low Risk In-Hospital Patients With Coronavirus Disease 2019 Infection. American Journal of Cardiology 2020; S0002-9149(20)31308-4.

^g^ Lim WS, van der Eerden MM, Laing R, et al. Defining community acquired pneumonia severity on presentation to hospital: an international derivation and validation study. Thorax. 2003;58(5):377-382. doi:10.1136/thorax.58.5.377

^h^ Guo J, Zhou B, Zhu M, et al. CURB-65 may serve as a useful prognostic marker in COVID-19 patients within Wuhan, China: a retrospective cohort study. Epidemiol Infect. 2020;148:e241. Published 2020 Oct 1. doi:10.1017/S0950268820002368

^i^ Fine MJ, Auble TE, Yealy DM, Hanusa BH, Weissfeld LA, Singer DE, Coley CM, Marrie TJ, Kapoor WN. A prediction rule to identify low-risk patients with community-acquired pneumonia. N Engl J Med. 1997 Jan 23;336(4):243-50. doi: 10.1056/NEJM199701233360402. PMID: 8995086.

^j^ Satici C, Demirkol MA, Sargin Altunok E, Gursoy B, Alkan M, Kamat S, Demirok B, Surmeli CD, Calik M, Cavus Z, Esatoglu SN. Performance of pneumonia severity index and CURB-65 in predicting 30-day mortality in patients with COVID-19. Int J Infect Dis. 2020 Sep;98:84-89. doi: 10.1016/j.ijid.2020.06.038. Epub 2020 Jun 14. PMID: 32553714; PMCID: PMC7293841.
